# Programmatically Selected Multidrug-Resistant Strains Drive the Emergence of Extensively Drug-Resistant Tuberculosis in South Africa

**DOI:** 10.1371/journal.pone.0070919

**Published:** 2013-08-23

**Authors:** Borna Müller, Violet N. Chihota, Manormoney Pillay, Marisa Klopper, Elizabeth M. Streicher, Gerrit Coetzee, Andre Trollip, Cindy Hayes, Marlein E. Bosman, Nicolaas C. Gey van Pittius, Thomas C. Victor, Sebastien Gagneux, Paul D. van Helden, Robin M. Warren

**Affiliations:** 1 DST/NRF Centre of Excellence for Biomedical Tuberculosis Research/MRC Centre for Molecular and Cellular Biology, Division of Molecular Biology and Human Genetics, Faculty of Medicine and Health Sciences, Stellenbosch University, Cape Town, South Africa; 2 Swiss Tropical and Public Health Institute, Basel, Switzerland; 3 University of Basel, Basel, Switzerland; 4 The Aurum Institute for Health Research, Johannesburg, South Africa; 5 Medical Microbiology and Infection Control, University of KwaZulu-Natal, Durban, South Africa; 6 National TB Reference Laboratory, National Institute for Communicable Diseases, National Health laboratory Services, Johannesburg, South Africa; 7 National Health Laboratory Service, Port Elizabeth, South Africa; 8 National Health Laboratory Service, Green Point, Cape Town, South Africa; Johns Hopkins University School of Medicine, United States of America

## Abstract

**Background:**

South Africa shows one of the highest global burdens of multidrug-resistant (MDR) and extensively drug-resistant (XDR) tuberculosis (TB). Since 2002, MDR-TB in South Africa has been treated by a standardized combination therapy, which until 2010 included ofloxacin, kanamycin, ethionamide, ethambutol and pyrazinamide. Since 2010, ethambutol has been replaced by cycloserine or terizidone. The effect of standardized treatment on the acquisition of XDR-TB is not currently known.

**Methods:**

We genetically characterized a random sample of 4,667 patient isolates of drug-sensitive, MDR and XDR-TB cases collected from three South African provinces, namely, the Western Cape, Eastern Cape and KwaZulu-Natal. Drug resistance patterns of a subset of isolates were analyzed for the presence of commonly observed resistance mutations.

**Results:**

Our analyses revealed a strong association between distinct strain genotypes and the emergence of XDR-TB in three neighbouring provinces of South Africa. Strains predominant in XDR-TB increased in proportion by more than 20-fold from drug-sensitive to XDR-TB and accounted for up to 95% of the XDR-TB cases. A high degree of clustering for drug resistance mutation patterns was detected. For example, the largest cluster of XDR-TB associated strains in the Eastern Cape, affecting more than 40% of all MDR patients in this province, harboured identical mutations concurrently conferring resistance to isoniazid, rifampicin, pyrazinamide, ethambutol, streptomycin, ethionamide, kanamycin, amikacin and capreomycin.

**Conclusions:**

XDR-TB associated genotypes in South Africa probably were programmatically selected as a result of the standard treatment regimen being ineffective in preventing their transmission. Our findings call for an immediate adaptation of standard treatment regimens for M/XDR-TB in South Africa.

## Introduction

The emergence of multidrug-resistant (MDR) and extensively drug-resistant (XDR) tuberculosis (TB) threatens disease control efforts throughout the world [1]–[3]. Drug-resistant TB may be acquired if bacteria harbouring spontaneously emerging drug resistance mutations (Table 1) are positively selected due to e.g. inadequate treatment regimens, poor drug quality or patient non-compliance [2], [4]–[6]. Alternatively, drug-resistant TB may also occur through the transmission of already resistant strains; termed primary resistance. High rates of primary resistance reflect, poor transmission control essentially due to delays in drug susceptibility testing and initiation of appropriate treatment [2], [5].

**Table 1:** Drug resistance-associated genetic regions analyzed.

Genetic region	Region covered*	No. of base-pairs	Resistance
*katG* gene	2154968…2155387	420	H
*inhA* promoter	1673261…1673506	246	H, Eto
*rpoB* gene	760822…761258	437	R
*embB* gene	4247302…4247561	260	E
*pncA* gene	2288652…2289266	615	Z
*rrs* gene (around nucleotide position 513)	1472283…1472852	570	S
*rrs* gene (around nucleotide position 1401)	1473184…1473373	190	Km, Am, Cm
*gyrA* gene	7355…7698	344	Many FQs, e.g. Ofx

* Genetic region covered by PCR with respect to nucleotide positions in H37Rv.

H: Isoniazid.

Eto: Ethionamide.

R: Rifampicin.

E: Ethambutol.

Z: Pyrazinamid.

S: Streptomycin.

Km: Kanamycin.

Am: Amikacin.

Cm: Capreomycin.

FQ: Fluoroquinolone.

Ofx: Ofloxacin.

Globally, in 2011, there were an estimated 310,000 incident cases of MDR-TB among cases reported to have tuberculosis of which 9% were XDR-TB [3], [4]. Increasing incidence rates for MDR-TB were recorded in several settings with South Africa being among the most severely affected countries [1], [7], [8]. In South Africa, 10% of all TB cases are believed to be MDR-TB of which again one-tenth are XDR-TB [1], [7], [8]. Highest rates of MDR and XDR-TB were notified for the Western Cape, Eastern Cape and KwaZulu-Natal provinces [9] with treatment success rates below 50% for MDR-TB and considerably poorer outcomes for XDR-TB [10], [11]. There is convincing evidence that MDR-TB in South Africa is caused mostly by the transmission of MDR strains, as suggested by well-documented clonal outbreaks and elevated rates of primary resistance (in some places as high as 80%) among MDR-TB cases [12]–[17]. Similarly, transmission of MDR strains is likely to be a main driver of MDR-TB in many other high-burden countries [2], [5], [18].

New TB patients in South Africa are treated according to WHO guidelines with isoniazid (H), rifampicin (R), ethambutol (E) and pyrazinamide (Z) [19]. Since 2002, MDR-TB treatment is also standardized and until 2010 included a fluoroquinolone (FQ; mostly ofloxacine [Ofx]), kanamycin (Km), ethionamide (Eto), E and Z [20]. This regimen neglected high proportions of E and Z resistance among MDR-TB cases and cross-resistance to Eto if infecting strains previously acquired an *inhA* promoter mutation (Table 1) [21], [22]. An only marginally improved MDR-TB regimen was implemented in 2010, which replaced E with cycloserine or terizidone (Cs/Trd) [20]. Standardized chemotherapy for MDR-TB is necessary in resource-limited settings where drug susceptibility testing (DST) cannot be performed regularly [19]. The design of standardized regimens however, requires the prior determination of the spectrum of resistances present in the community [19]. Culture-based resistance surveys not incorporating strain genotyping data do not enable examining whether detected resistances are transmitted jointly (by the same strain) or independently (by different strains). The absence of this knowledge has important implications for the design of standardized treatment regimens.

Previous studies in South Africa observed an association of specific genotypes of *M. tuberculosis* with XDR-TB [23]. Specifically, the R220 genotype, a subgroup of the typical Beijing family of strains, the R86 genotype, a subgroup of “atypical” Beijing strains and the F15/LAM4/KZN genotype, a subgroup of the LAM4 family, were identified as commonly transmitted drug-resistant strains in the Western Cape, Eastern Cape and KwaZulu-Natal, respectively [15], [16], [23]–[26]. In order to elucidate whether and how standardized treatment impacted the strain population structure of drug-sensitive and drug-resistant *M. tuberculosis* in South Africa, we characterized in detail an extensive collection of clinical TB isolates from these provinces and analyzed resistance patterns of XDR-TB associated strains.

## Materials and Methods

### Ethics statement

This study was approved by the Ethics Committees of Stellenbosch University and the University of KwaZulu-Natal. The Stellenbosch Health Research Ethics Committee approved a waiver of consent for the retrospective genotypic analysis of routinely collected *M. tuberculosis* isolates after patient identifiers were removed. The University of KwaZulu-Natal Ethic Committee approved the prospective collection and genotyping of *M. tuberculosis* isolates after obtaining written consent.

### Study population, routine culture and drug susceptibility testing

**Figure 1:**
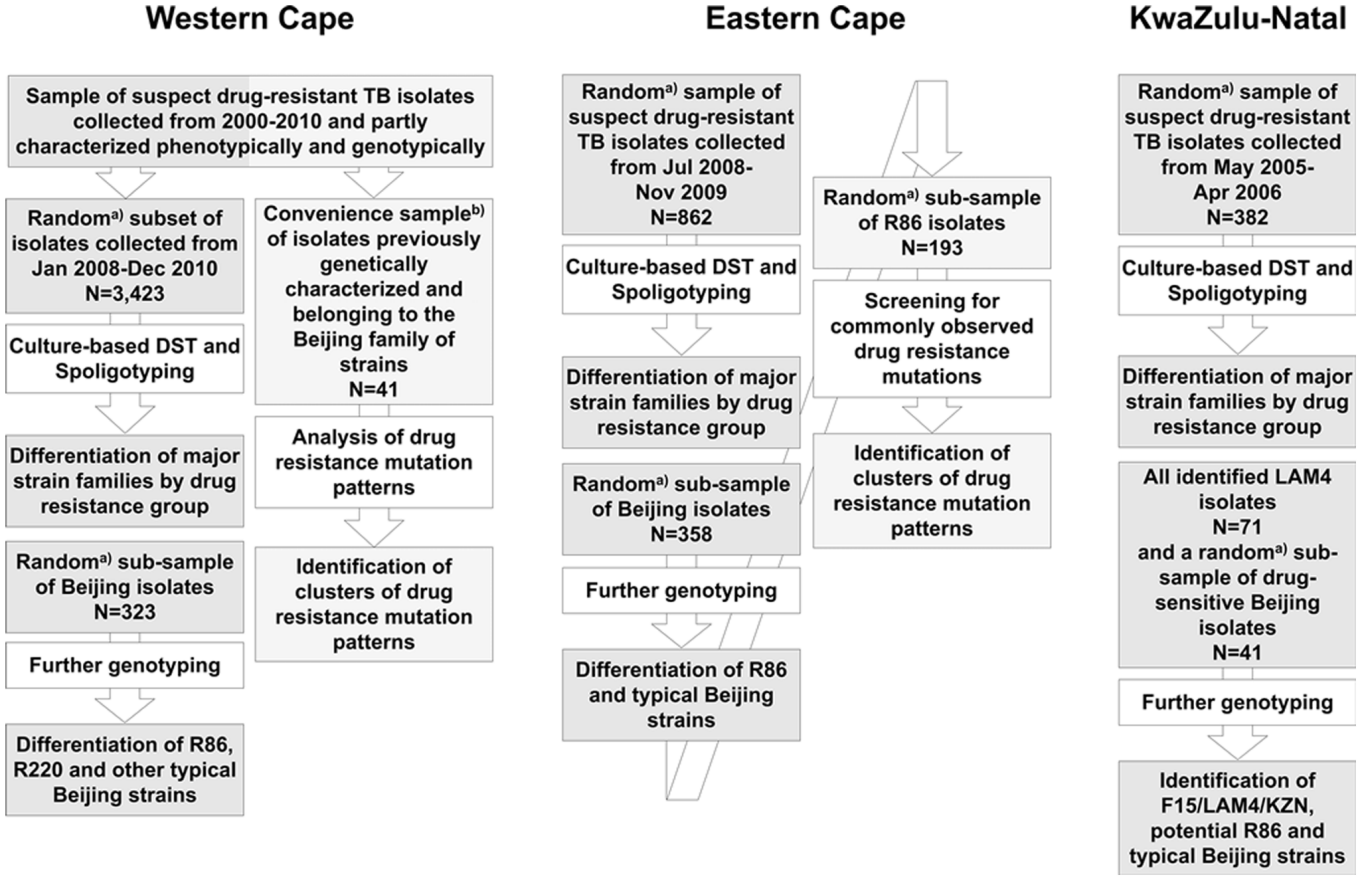
Selection of study population. Grey boxes indicate sample sets used to analyze the strain population structures in the three South African provinces. Boxes with striped pattern indicate sample sets used to characterize drug resistance mutation patterns among XDR-TB associated genotypes. ^a)^ Computer-based random sampling was applied. ^b)^ Review of an extensive collection of data generated within multiple previous studies.

A comprehensive sample of clinical drug-resistant TB isolates collected during different time periods from the whole area of the Western Cape, Eastern Cape and KwaZulu-Natal province were analysed (Figure 1). Only one isolate per patient was included in the study. Subsets of this sample collection were used previously to describe the population structure of MDR *M. tuberculosis* strains in these provinces [23] and drug resistance mutations of strains of the Eastern Cape Province [27]. These isolates characterised formerly were further complemented with a comparable, random sample of diagnosed drug-sensitive and mono-/poly-resistant isolates in order to analyse a larger spectrum of resistance patterns and a wider geographical area compared to previous studies. Routine culture and DST was performed at the National Health Laboratory Service (NHLS) in the respective provinces as described previously [23]. The location of healthcare facilities attended by the TB patients was recorded to analyze the geographical distribution of *M. tuberculosis* genotypes identified.

### Definition of drug resistance groups

*M. tuberculosis* isolates were classified into different drug resistance groups based on routine DST [23]. Drug-sensitive isolates were susceptible to all drugs tested (at least H and R). Mono-/Poly-resistant isolates were resistant to one or multiple first-line anti-TB drugs but were not MDR. MDR and XDR isolates were classified according to WHO definitions [19]. Pre-XDR-TB isolates were defined as MDR-TB isolates with additional resistance to either a FQ or a second-line injectable drug (Km, amikacin [Am] or capreomycin [Cm]) but not both. The MDR *sensu stricto* (*s.s.*) group excluded identified pre-XDR and XDR isolates from MDR isolates.

### Genotypic characterization

Initial genotyping of random samples of *M. tuberculosis* isolates was done by spoligotyping according to the protocol described by Kamerbeek *et al* [28] and the isolates were grouped into recognized strain families by comparison to previously reported spoligotype patterns [29],[30]. A randomly selected subset of Beijing isolates from all drug resistance groups from the Western and Eastern Cape and a subset of only drug-sensitive Beijing isolates from KwaZulu-Natal were further differentiated into typical and “atypical” Beijing isolates by PCR (Figure 1) [14]. Computer-based random sampling was applied to randomly select isolates. Based on similar IS*6110* RFLP patterns and whole genome sequencing data it was previously established that “atypical” Beijing strains in the Western and Eastern Cape represent one single genotype herein referred to as R86 [14], [25], [31]. Typical Beijing isolates from the Western Cape were distinguished into R220 and non-R220 isolates by PCR (Figure 1) [32]. LAM4 isolates from KwaZulu-Natal were differentiated into F15/LAM4/KZN and other LAM4 isolates by IS*6110* RFLP analysis (Figure 1) [16]. A random subsample of identified MDR R86 isolates from the Eastern Cape was tested for the presence of drug resistance mutations in the *inhA* promoter and the genes *katG*, *rpoB*, *pncA*, *embB*, *rrs* and *gyrA* by PCR amplification of genetic regions commonly observed to harbour resistance mutations and subsequent sequencing of these PCR products (Table 1, Figure 1) [33]–[37]. Similarly, data from an extensive collection of drug-resistant isolates from the Western Cape was reviewed for records on Beijing isolates tested for the presence of resistance mutations in the same genetic regions (Table 1, Figure 1). However, no data on streptomycin resistance mutations in *rrs* were available (Table 1). Isolates with identical drug resistance mutation patterns were grouped by *pncA* mutations, which are highly diverse and may allow identifying genetically related groups of strains [27].

## Results

**Figure 2:**
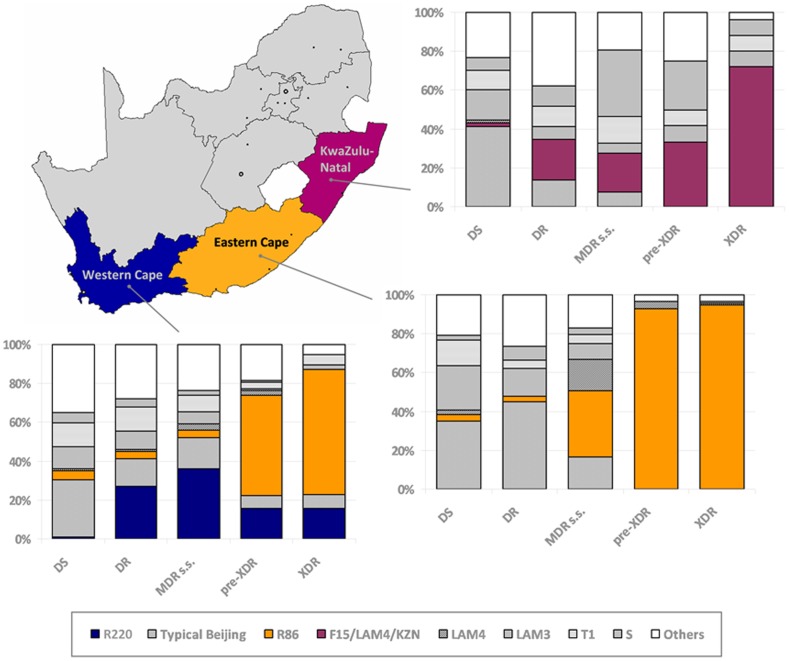
Strain population structure of drug-sensitive (DS), mono-/poly-resistant (DR), *sensu stricto* multidrug-resistant (MDR *s.s.*; excluding identified pre-XDR and XDR isolates), pre-extensively drug-resistant (pre-XDR) and extensively drug resistant (XDR) isolates in three provinces of South Africa. The R220, R86 and F15/LAM4/KZN genotypes, respectively, represent a subgroup of the typical Beijing, “atypical” Beijing and LAM4 family [14]–[16], [22]–[24]. Based on similar IS*6110* RFLP patterns and whole genome sequencing data it was previously shown that “atypical” Beijing strains in the Western and Eastern Cape, unlike in other parts of the world, represent one single genotype herein referred to as R86 [23], [25], [27]. The specific presence of R220 and F15/LAM4/KZN genotypes was only assessed in the Western Cape and KwaZulu-Natal, respectively, where these genotypes were known to be frequent among XDR-TB cases [22].

Molecular characterization of a random sample of 4,667 clinical TB isolates collected from the whole area of the Western Cape, Eastern Cape and KwaZulu-Natal provinces of South Africa revealed an increasing predomination of a single genotype of strains from drug-sensitive to XDR-TB, in each of the three provinces (Figure 2). In the Eastern Cape and KwaZulu-Natal, the proportion of isolates belonging to the R86 and the F15/LAM4/KZN genotype, respectively, underwent a 27- and 44-fold increase from drug-susceptible to XDR-TB and accounted for 95% and 72% of all XDR-TB cases (Figure 2, Table S1). In the Western Cape, the percentage of R86 isolates also increased significantly from drug-sensitive to XDR-TB cases. However, a previous study indicated that R86 isolates detected in the Western Cape, may to a large extent represent TB patients from the economically depressed Eastern Cape seeking treatment in the more affluent Western Cape [23]. Thus, if R86 isolates are disregarded, the R220 genotype most strongly contributes to drug-resistant TB in the Western Cape, in line with previous results [15]. Noteworthy, R220 isolates expand significantly in proportion (24-fold) from drug-sensitive to mono-/poly-resistant TB (Figure 2, Table S1).

Genotypes predominant in XDR-TB were infrequently detected among drug-sensitive TB cases (Figure 2). In all three provinces investigated, R220, R86 and F15/LAM4/KZN strains accounted for less than 5% of the drug-sensitive TB cases, making them considerably less abundant than the typical Beijing, LAM3 and T1 genotypes, which each represented between 10% and 41% of all drug-sensitive isolates (Table S1). Interestingly, while the strain population structure among MDR-TB isolates was fundamentally different between the three provinces [23], it appeared to be similar for drug-sensitive isolates (Figure 2).

**Figure 3:**
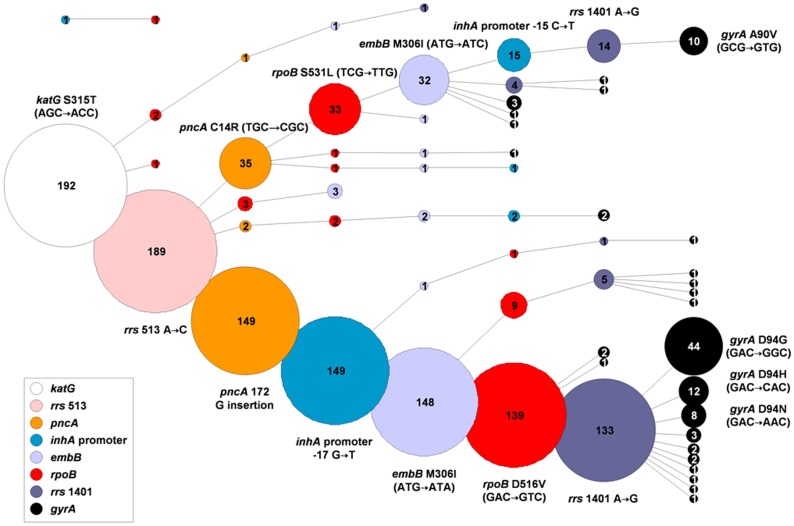
Drug resistance mutation pattern in a random selection of 193 MDR R86 isolates from the Eastern Cape. Different colours indicate different drug resistance associated genes. The area of the circles is proportional to the number of isolates (indicated in the centre of each circle) harbouring an identical drug resistance mutation for the respective resistance gene as well as all circles connected to the left. Principal branches of the tree were defined by resistance mutations in *pncA*. Other first-line drug resistance mutations were connected by logical deduction to maximize clustering and were followed by second-line resistance mutations. However, the order of acquisition of resistance mutations may remain debatable in some cases.

**Figure 4:**
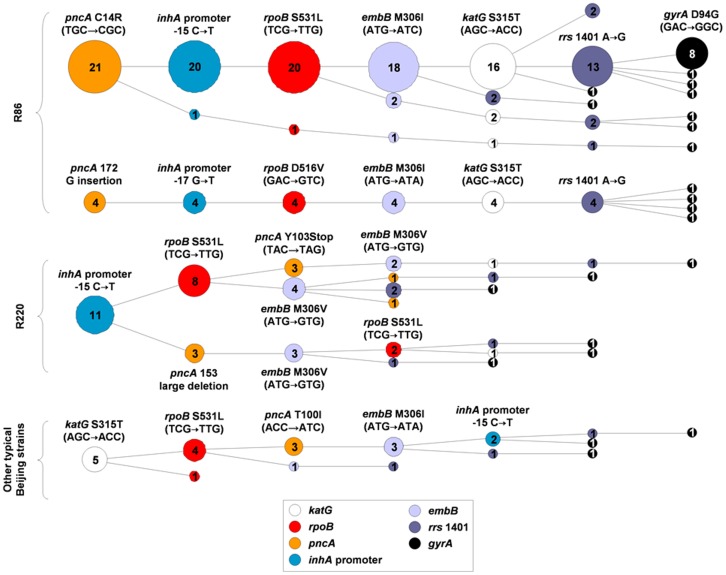
Drug resistance mutation pattern in a convenience sample of 41 MDR Beijing isolates from the Western Cape. No data was available for the streptomycin resistance determining region in *rrs* (Table 1). For more information see figure legend of Figure 3.

Drug resistance patterns of XDR-TB associated genotypes were analysed by assessing the presence of commonly observed resistance mutations in the *inhA* promoter and the genes *katG*, *rpoB*, *pncA*, *embB*, *rrs* and *gyrA* (Table 1). A random sample of 193 MDR isolates of the R86 genotype from the Eastern Cape and 41 conveniently selected MDR isolates from the Western Cape representing a variety of different Beijing genotypes (R86, R220 and other typical Beijing strains) were selected (Figures 1, 3 and 4). Apart from H and R resistance mutations, various additional resistance-conferring mutations were detected. Moreover, mutation patterns were highly clustered (Figures 3 and 4). Most strikingly, 69% of the R86 isolates from the Eastern Cape analyzed (133/193 MDR isolates analyzed) harboured as many as seven identical resistance mutations in the *inhA* promoter and the genes *katG*, *rpoB*, *pncA*, *embB* and *rrs* suggesting that this cluster represents a commonly transmitted pre-XDR strain resistant to at least H, R, Z, E, S, Eto, Km, amikacin (Am) and capreomycin (Cm) (Table 1, Figure 3) [23], [27]. XDR-TB cases that have emerged from infection with this strain showed a variety of different *gyrA* mutations, suggesting that FQ resistance was acquired subsequently and perhaps due to the mismanagement of primary pre-XDR-TB. Nevertheless, a sub-group of 44 isolates showed for example an identical *gyrA* D94G mutation, potentially indicating community spread of XDR strains (Figure 3).

A second cluster representing 17% of the R86 isolates from the Eastern Cape (32/193 MDR isolates analyzed), was characterized by identical mutations in *katG*, *rpoB*, *pncA*, *embB* and *rrs* conferring resistance to H, R, Z, E and S (Table 1; Figure 3) [27]. Presumably, a sub-branch of this strain subsequently acquired resistance to Eto through an *inhA* promoter mutation [38], to Km, Am and Cm through an additional mutation in *rrs* [39], [40] and finally to FQs due to the acquisition of a *gyrA* A90V resistance mutation (Figure 3) [6], [41].

Analysis of the drug resistance mutation patterns of a convenience sample of 41 MDR Beijing isolates from the Western Cape, revealed that the two major R86 clusters detected in the Eastern Cape were also present in this province, albeit at a different relative frequency (Figures 3 and 4). For the remaining R220 and other typical Beijing isolates analyzed, clustered mutation patterns for at least *pncA* and *embB* were found in 8 out of 16 cases (Figure 4), indicating a widespread combined presence of Z and E resistance among these strains, in the Western Cape.

The clusters of strains defined above by genotype and drug resistance mutation patterns (Figures 3 and 4) were geographically widespread within the Eastern and Western Cape (Table 2), indicating historical spread. In the Eastern Cape, the two predominant clusters among MDR isolates of the R86 genotype were detected in four and three different municipal districts, respectively. In the Western Cape, despite the small sample size, isolates of four out of five clusters as defined by distinct *pncA* mutations were identified in more than one district (Table 2).

**Table 2:** Geographical distribution of selected clusters of isolates.

Province	Genotype	Drug resistance mutation pattern	Municipal District	N_Isolate_	%
EC	R86	*katG* S315T/*rrs* 513 A→C/*pncA* C14R/*rpoB* S531L/*embB* M306I	Amathole	19	59.4
			Nelson Mandela Bay	12	37.5
			OR Tambo	1	3.1
EC	R86	*katG S*315T/*rrs 5*13 A→C/*pncA* C14R/*rpoB* S531L/*embB* M306I/*inhA* promoter -15 C→T/*rrs* 1401 A→G/*gyrA* A90V	Amathole	7	70.0
			Nelson Mandela Bay	2	20.0
			OR Tambo	1	10.0
EC	R86	*katG* S315T/*rrs* 513 A→C/*pncA* 172 G insertion/*inhA* promoter -17 G→T/*embB* M306I/*rpoB* D516V/*rrs* 1401 A→G	Amathole	30	22.6
			Cacadu	12	9.0
			Chris Hani	1	0.8
			Nelson Mandela Bay	90	67.7
EC	R86	*katG* S315T/*rrs* 513 A→C/*pncA* 172 G insertion/*inhA* promoter -17 G→T/*embB* M306I/*rpoB* D516V/*rrs* 1401 A→G/*gyrA* D94G	Amathole	9	20.5
			Cacadu	3	6.8
			Chris Hani	1	2.3
			Nelson Mandela Bay	31	70.5
WC	R86	*pncA* C14R/*inhA* promoter -15 C→T/*rpoB* S531L/*embB* M306I/*katG* S315T/*rrs* 1401 A→G	Cape Town	12	92.3
			Eden	1	7.7
WC	R86	*pncA* 172 G insertion/*inhA* promoter -17 G→T/*rpoB* D516V/*embB* M306I/*katG* S315T/*rrs* 1401 A→G	Cape Town	3	75.0
			Eden	1	25.0
WC	R220	*inhA* promoter -15 C→T/*rpoB* S531L/*pncA* Y103Stop	Cape Town	2	66.7
			Overberg	1	33.3
WC	R220	*inhA* promoter -15 C→T/*pncA* 153 large deletion/*embB* M306V	Cape Town	2	66.7
			Cape Winelands	1	33.3
WC	Other typical Beijing	*katG* S315T/*rpoB* S531L/*pncA* T100I/*embB* M306I	Cape Town	3	100.0

EC: Eastern Cape Province.

WC: Western Cape Province.

N_isolate_: Number of isolates of a cluster detected in the municipal district indicated.

%: Proportion of isolates of a cluster detected in the municipal district indicated.

## Discussion

The present data shows a strong association between distinct strain genotypes and the emergence of XDR-TB in three neighbouring provinces of South Africa [23]. XDR-TB associated genotypes were infrequently found among drug-sensitive TB cases, of which typical Beijing, LAM3 and T1 were the most prevalent genotypes in all three provinces (Figure 2). This observation is counterintuitive, if it was supposed that the proportion of genotypes causing XDR-TB was a result of random fluctuations. Under such conditions it would be plausible to assume that genotypes predominant among drug-sensitive TB cases would have been more likely to become overrepresented among XDR-TB cases (Table 2, Figure 2). Instead, the association of the R220, R86 and F15/LAM4/KZN genotypes with XDR-TB suggests an increased ability of these strains to acquire multiple drug-resistance mutations or to transmit as drug-resistant strains. However, the relatively distant phylogenetic relationship of these XDR-TB associated strain genotypes [42], [43] argues against the possibility of genetic background accounting for this observation.

Drug resistance mutation patterns of isolates of XDR-TB associated genotypes in the Eastern and Western Cape provinces were highly clustered (Figure 3). Unfortunately, isolates of the XDR-TB associated F15/LAM4/KZN genotype in KwaZulu-Natal were not further characterized within this study and therefore the relationship between genotype and clustering could not be evaluated. However, in line with our observations for the Western and Eastern Cape, a previous whole genome sequence analysis of nine XDR F15/LAM4/KZN isolates from patients of different settings in KwaZulu-Natal revealed nearly identical genome sequences including matching drug resistance mutations [26]. Together, this data suggests that in South Africa, XDR-TB emerges mainly due to ongoing transmission of specific MDR *s.s.* or pre-XDR genotypes that are sub-optimally treated by programmatic treatment regimens, or partly, directly through the transmission of XDR strains of these genotypes [11], [25], [27].

It is likely however, that our analyses convey a relative overestimate of the proportion of transmission of primary pre-XDR and XDR strains as the Km/Am/Cm resistance mutation (*rrs* 1401 A→G) and the FQ resistance mutations (*gyrA* D94G and the *gyrA* A90V) detected among the largest clusters of isolates, belong to the most frequently observed resistance mutations for these drugs [6], [44]. Indeed, for the Km/Am/Cm resistance mutations observed in *rrs*, only a very low diversity was observed (Figures 3 and 4) [39], [40]. Thus, it is likely that these mutations have been acquired independently multiple times among clustered isolates and clustering may not (or to a lesser extent) represent the clonal spread of pre-XDR and XDR strains.

Even if FQ and Km/Am/Cm resistance mutations in *gyrA* and *rrs* are disregarded, 72% (139/193) and 8% (15/193) of the MDR R86 isolates from the Eastern Cape belonged to one of two major clusters of isolates harbouring identical resistance mutations to at least H, R, Z, E, S and Eto (Figure 3). Similarly, altogether 63% (26/41) of the MDR Beijing isolates from the Western Cape tested belonged to one of altogether five clusters of isolates with identical resistance mutations to at least H, R, Z, E, and Eto (Figure 4). Given this data and the frequency distribution of different genotypes among MDR-TB cases (Table 2), we can estimate that at least 48% and 28% of all MDR-TB cases in the Eastern and Western Cape, respectively, were caused by a strain resistant to at least H, R, Z, E, S and Eto at the time of infection. Considering published whole genome sequences of XDR F15/LAM4/KZN isolates [26] and if FQ and Km/Am/Cm resistance mutations are disregarded, this genotype also shows primary resistance to at least H, R, E, Z, S and Eto and accounts for 26% of all MDR-TB cases in KwaZulu-Natal (Table 2). Importantly, since only specific XDR-TB associated genotypes were analyzed, the proportion of MDR-TB cases with resistances to additional anti-TB drugs than H and R may be even higher.

Given the standard MDR-TB drug regimens in South Africa (currently consisting of Ofx, Km, Eto, Trd/Cs and Z) and if excluding *rrs* and *gyrA* mutations, TB patients infected with these strains are exposed to three effective drugs only (Ofx, Km and Cs/Trd); this is less than the four effective drugs recommended by the WHO [45]. If many of these transmitting strains in fact also harboured a primary *rrs* 1401 A→G mutation, the treatment regimen would consist of two effective drugs only. Under these conditions, even the standardized XDR-TB treatment regimen in South Africa, currently consisting of moxifloxacin, Cm, Eto, para-aminosalicylic acid and Cs/Trd would be inappropriate to treat infected patients [20]. Noteworthy, the previous MDR-TB regimen endorsed until 2010, which used E instead of Cs/Trd, resulted in an even higher chance of resistance development as it consisted of only two or one effective drug, respectively. This clearly demonstrates the inadequacy of current treatment regimens in South Africa to prevent spread of XDR-TB associated strains and calls for an immediate adaptation of MDR treatment algorithms. Moreover, our findings highlight the urgent need for rapid first- and second-line DST for all TB cases at treatment onset.

**Figure 5:**
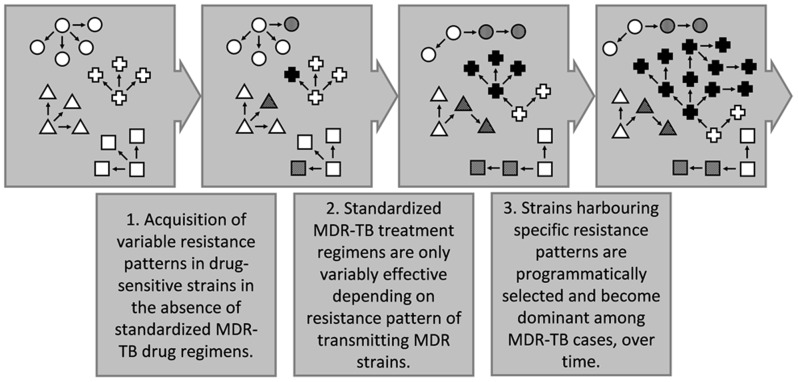
Model for the evolution of XDR-TB associated strain families.

A likely scenario for the evolution of XDR-TB associated strains in South Africa is depicted in Figure 5. It could be speculated that the use of non-standardized drug regimens before 2002 facilitated the emergence and transmission of strains with different resistance patterns. Possibly, the implementation of standardized MDR-TB treatment subsequently promoted the spread of strains harbouring resistances against which the regimen was less effective. These strains could have emerged originally as early as in the 1950's when TB treatment was not well controlled and mostly included H, S and para-aminosalicylic acid only [46]. This is supported by the very widespread presence of identical H and S resistance mutations in isolates from the Eastern Cape, indicating that these mutations were acquired at an initial stage (Figure 3). However, importantly, improved TB control and standardized MDR-TB treatment probably curbed the emergence of new resistant strains and transmission of strains harbouring unfavourable resistance patterns. Thus, the programmatic use of an only variably effective MDR-TB treatment regimen could explain the predomination of only a few strain families among XDR-TB cases. Although an impact of strain genetic background on the propensity to develop MDR/XDR-TB has been suggested [47], according to this model, the acquisition of advantageous resistance patterns would have occurred by chance and independent of strain genetic background, explaining the association of different, distantly related genotypes with XDR-TB in different provinces. Associations of a few specific genotypes with MDR and XDR-TB were observed in several countries throughout the world [48]–[51], suggesting similar mechanisms for the emergence of XDR-TB.

This work highlights the value of molecular epidemiological tools to perform drug resistance surveys and to decipher how individual resistances may be linked and transmitted. Moreover, this data will help designing more effective and urgently needed MDR-TB treatment regimens for South Africa. Failure to do so will rapidly enhance spread and amplification of resistance among XDR-TB associated strains.

## Supporting Information

Table S1 **Strain population structure in the Western Cape, Eastern Cape and KwaZulu-Natal Provinces of South Africa.**
(XLS)

## References

[1] WHO (2010) Multidrug and extensively drug-resistant TB (M/XDR-TB) 2010 Global report on surveillance and response. Available: http://whqlibdoc.who.int/publications/2010/9789241599191_eng.pdf. Accessed 2012 Jun 11.

[2] Gandhi NR, Nunn P, Dheda K, Schaaf HS, Zignol M, et al (2010) Multidrug-resistant and extensively drug-resistant tuberculosis: a threat to global control of tuberculosis. Lancet 375: 1830–1843. 20488523 10.1016/S0140-6736(10)60410-2

[3] WHO (2012) Global Tuberculosis Report 2012. Available: http://apps.who.int/iris/bitstream/10665/75938/1/9789241564502_eng.pdf. Accessed 2013 Jan 23.

[4] Zumla A, Raviglione M, Hafner R, von Reyn CF (2013) Tuberculosis. N Engl J Med 368: 745–755. 23425167 10.1056/NEJMra1200894

[5] Muller B, Warren RM, Williams M, Bottger E, Gey Van Pittius NC, et al.. (2012) Acquisition, Transmission and Amplification of Drug-Resistant Tuberculosis. In: Donald PR, van Helden PD, editors. Progress in Respiratory Research. Karger. pp. 96–104.

[6] Sandgren A, Strong M, Muthukrishnan P, Weiner BK, Church GM, et al (2009) Tuberculosis drug resistance mutation database. PLoS Med 6: e2. 19209951 10.1371/journal.pmed.1000002 PMC2637921

[7] Zignol M, Van GW, Falzon D, Sismanidis C, Glaziou P, et al (2012) Surveillance of anti-tuberculosis drug resistance in the world: an updated analysis, 2007–2010. Bull World Health Organ 90: 111–119D. 22423162 10.2471/BLT.11.092585 PMC3302554

[8] WHO-IUTALD Global Project on anti-tuberculosis drug resistance surveillance (2008) Ant-tuberculosis drug resistance in the world (Report No 4). Available: http://www.who.int/tb/publications/2008/drs_report4_26feb08.pdf. Accessed 2012 Jun 6.

[9] National Health Laboratory Services (2010) National Institute for Communicable Diseases - Annual Report 2009. Available: http://www.nicd.ac.za/assets/files/Annual_report_2009.pdf. Accessed 2012 Jun 1.

[10] Farley JE, Ram M, Pan W, Waldman S, Cassell GH, et al (2011) Outcomes of multi-drug resistant tuberculosis (MDR-TB) among a cohort of South African patients with high HIV prevalence. PLoS One 6: e20436. 21799728 10.1371/journal.pone.0020436 PMC3142109

[11] Dheda K, Shean K, Zumla A, Badri M, Streicher EM, et al (2010) Early treatment outcomes and HIV status of patients with extensively drug-resistant tuberculosis in South Africa: a retrospective cohort study. Lancet 375: 1798–1807. 20488525 10.1016/S0140-6736(10)60492-8

[12] van Rie A, Warren RM, Beyers N, Gie RP, Classen CN, et al (1999) Transmission of a multidrug-resistant *Mycobacterium tuberculosis* strain resembling “strain W” among noninstitutionalized, human immunodeficiency virus-seronegative patients. J Infect Dis 180: 1608–1615. 10515823 10.1086/315054

[13] Victor TC, Streicher EM, Kewley C, Jordaan AM, van der Spuy GD, et al (2007) Spread of an emerging *Mycobacterium tuberculosis* drug-resistant strain in the western Cape of South Africa. Int J Tuberc Lung Dis 11: 195–201. 17263291

[14] Strauss OJ, Warren RM, Jordaan A, Streicher EM, Hanekom M, et al (2008) Spread of a low-fitness drug-resistant *Mycobacterium tuberculosis* strain in a setting of high human immunodeficiency virus prevalence. J Clin Microbiol 46: 1514–1516. 18272712 10.1128/JCM.01938-07 PMC2292903

[15] Johnson R, Warren RM, van der Spuy GD, Gey Van Pittius NC, Theron D, et al (2010) Drug-resistant tuberculosis epidemic in the Western Cape driven by a virulent Beijing genotype strain. Int J Tuberc Lung Dis 14: 119–121. 20003705

[16] Pillay M, Sturm AW (2007) Evolution of the extensively drug-resistant F15/LAM4/KZN strain of *Mycobacterium tuberculosis* in KwaZulu-Natal, South Africa. Clin Infect Dis 45: 1409–1414. 17990220 10.1086/522987

[17] Cox HS, McDermid C, Azevedo V, Muller O, Coetzee D, et al (2010) Epidemic levels of drug resistant tuberculosis (MDR and XDR-TB) in a high HIV prevalence setting in Khayelitsha, South Africa. PLoS One 5: e13901. 21085569 10.1371/journal.pone.0013901 PMC2981525

[18] Zhao Y, Xu S, Wang L, Chin DP, Wang S, et al (2012) National survey of drug-resistant tuberculosis in China. N Engl J Med 366: 2161–2170. 22670902 10.1056/NEJMoa1108789

[19] WHO (2010) Treatment of Tuberculosis Guidelines. Available: http://www.who.int/tb/publications/tb_treatmentguidelines/en/index.html. Accessed 2013 Mar 28.

[20] Streicher EM, Muller B, Chihota V, Mlambo C, Tait M, et al (2012) Emergence and treatment of multidrug resistant (MDR) and extensively drug-resistant (XDR) tuberculosis in South Africa. Infect Genet Evol 12: 686–694. 21839855 10.1016/j.meegid.2011.07.019

[21] Hoek KGP, Schaaf HS, Van Pittius NCG, Van Helden PD, Warren RM (2009) Resistance to pyrazinamide and ethambutol compromises MDR/XDR-TB treatment. S Afr Med J 99: 785–787. 20218473

[22] Muller B, Streicher EM, Hoek KG, Tait M, Trollip A, et al (2011) *inhA* promoter mutations: a gateway to extensively drug-resistant tuberculosis in South Africa? Int J Tuberc Lung Dis 15: 344–351. 21333101

[23] Chihota VN, Muller B, Mlambo CK, Pillay M, Tait M, et al (2012) Population structure of multi- and extensively drug-resistant *Mycobacterium tuberculosis* strains in South Africa. J Clin Microbiol 50: 995–1002. 22170931 10.1128/JCM.05832-11 PMC3295122

[24] Gandhi NR, Moll A, Sturm AW, Pawinski R, Govender T, et al (2006) Extensively Drug Resistant Tuberculosis as a cause of death in patients co-infected with Tuberculosis and HIV in a rural area of South Africa. Lancet 368: 1575–1580. 17084757 10.1016/S0140-6736(06)69573-1

[25] Ioerger TR, Feng Y, Chen X, Dobos KM, Victor TC, et al (2010) The non-clonality of drug resistance in Beijing-genotype isolates of *Mycobacterium tuberculosis* from the Western Cape of South Africa. BMC Genomics 11: 670. 21110864 10.1186/1471-2164-11-670 PMC3091785

[26] Ioerger TR, Koo S, No EG, Chen X, Larsen MH, et al (2009) Genome analysis of multi- and extensively-drug-resistant tuberculosis from KwaZulu-Natal, South Africa. PLoS One 4: e7778. 19890396 10.1371/journal.pone.0007778 PMC2767505

[27] Klopper M, Warren RM, Hayes C, Gey van Pittius NC, Streicher E, et al (2013) Emergence and spread of Extensively and Totally Drug Resistant Tuberculosis in South Africa. Emerg Infect Dis 19: 449–455. 23622714 10.3201//EID1903.120246 PMC3647643

[28] Kamerbeek J, Schouls L, Kolk A, van Agterveld M, van Soolingen D, et al (1997) Simultaneous detection and strain differentiation of *Mycobacterium tuberculosis* for diagnosis and epidemiology. J Clin Microbiol 35: 907–914. 9157152 10.1128/jcm.35.4.907-914.1997 PMC229700

[29] Streicher EM, Victor TC, van der SG, Sola C, Rastogi N, et al (2007) Spoligotype signatures in the *Mycobacterium tuberculosis* complex. J Clin Microbiol 45: 237–240. 17065260 10.1128/JCM.01429-06 PMC1828946

[30] Brudey K, Driscoll JR, Rigouts L, Prodinger WM, Gori A, et al (2006) Mycobacterium tuberculosis complex genetic diversity: mining the fourth international spoligotyping database (SpolDB4) for classification, population genetics and epidemiology. BMC Microbiol 6: 23. 16519816 10.1186/1471-2180-6-23 PMC1468417

[31] Hanekom M, van der Spuy GD, Streicher E, Ndabambi SL, McEvoy CR, et al (2007) A recently evolved sublineage of the *Mycobacterium tuberculosis* Beijing strain family is associated with an increased ability to spread and cause disease. J Clin Microbiol 45: 1483–1490. 17360841 10.1128/JCM.02191-06 PMC1865897

[32] Johnson R, Warren RM, Strauss OJ, Jordaan A, Falmer AA, et al (2006) An outbreak of drug resistant Tuberculosis caused by a Beijing strain in the Western Cape, South Africa. Int J Tuberc Lung Dis 10: 1412–1414. 17167961

[33] Victor TC, Jordaan AM, van Rie A, van der Spuy GD, Richardson M, et al (1999) Detection of mutations in drug resistance genes of *Mycobacterium tuberculosis* by a dot-blot hybridization strategy. Tuber Lung Dis 79: 343–348. 10694978 10.1054/tuld.1999.0222

[34] Johnson R, Jordaan AM, Pretorius L, Engelke E, van der SG, et al (2006) Ethambutol resistance testing by mutation detection. Int J Tuberc Lung Dis 10: 68–73. 16466040

[35] Louw GE, Warren RM, Donald PR, Murray MB, Bosman M, et al (2006) Frequency and implications of pyrazinamide resistance in managing previously treated tuberculosis patients. Int J Tuberc Lung Dis 10: 802–807. 16848344

[36] Streicher EM, Bergval I, Dheda K, Bottger EC, Gey Van Pittius NC, et al (2012) *Mycobacterium tuberculosis* population structure determines the outcome of genetics-based second-line drug resistance testing. Antimicrob Agents Chemother 56: 2420–2427. 22330913 10.1128/AAC.05905-11 PMC3346650

[37] Sirgel FA, Tait M, Warren RM, Streicher EM, Bottger EC, et al (2012) Mutations in the *rrs* A1401G gene and phenotypic resistance to amikacin and capreomycin in *Mycobacterium tuberculosis* . Microb Drug Resist 18: 193–197. 21732736 10.1089/mdr.2011.0063

[38] Morlock GP, Metchock B, Sikes D, Crawford JT, Cooksey RC (2003) *ethA*, *inhA*, and *katG* loci of ethionamide-resistant clinical *Mycobacterium tuberculosis* isolates. Antimicrob Agents Chemother 47: 3799–3805. 14638486 10.1128/AAC.47.12.3799-3805.2003 PMC296216

[39] Georghiou SB, Magana M, Garfein RS, Catanzaro DG, Catanzaro A, et al (2012) Evaluation of genetic mutations associated with *Mycobacterium tuberculosis* resistance to amikacin, kanamycin and capreomycin: a systematic review. PLoS One 7: e33275. 22479378 10.1371/journal.pone.0033275 PMC3315572

[40] Jugheli L, Bzekalava N, de RP, Fissette K, Portaels F, et al (2009) High level of cross-resistance between kanamycin, amikacin, and capreomycin among *Mycobacterium tuberculosis* isolates from Georgia and a close relation with mutations in the *rrs* gene. Antimicrob Agents Chemother 53: 5064–5068. 19752274 10.1128/AAC.00851-09 PMC2786337

[41] Sirgel FA, Warren RM, Streicher EM, Victor TC, van Helden PD, et al (2012) *gyrA* mutations and phenotypic susceptibility levels to ofloxacin and moxifloxacin in clinical isolates of *Mycobacterium tuberculosis* . J Antimicrob Chemother 67: 1088–1093. 22357804 10.1093/jac/dks033

[42] Filliol I, Motiwala AS, Cavatore M, Qi W, Hazbon MH, et al (2006) Global phylogeny of *Mycobacterium tuberculosis* based on single nucleotide polymorphism (SNP) analysis: insights into tuberculosis evolution, phylogenetic accuracy of other DNA fingerprinting systems, and recommendations for a minimal standard SNP set. J Bacteriol 188: 759–772. 16385065 10.1128/JB.188.2.759-772.2006 PMC1347298

[43] Gagneux S, Small PM (2007) Global phylogeography of *Mycobacterium tuberculosis* and implications for tuberculosis product development. Lancet Infect Dis 7: 328–337. 17448936 10.1016/S1473-3099(07)70108-1

[44] Zhang Y, Yew WW (2009) Mechanisms of drug resistance in *Mycobacterium tuberculosis* . Int J Tuberc Lung Dis 13: 1320–1330. 19861002

[45] Caminero JA, Sotgiu G, Zumla A, Migliori GB (2010) Best drug treatment for multidrug-resistant and extensively drug-resistant tuberculosis. Lancet Infect Dis 10: 621–629. 20797644 10.1016/S1473-3099(10)70139-0

[46] Porteous JB (1959) The treatment of pulmonary tuberculosis. S Afr Med J 33: 265–267. 13646880

[47] Muller B, Borrell S, Rose G, Gagneux S (2013) The heterogeneous evolution of multidrug-resistant *Mycobacterium tuberculosis* . Trends Genet 29: 160–169. 23245857 10.1016/j.tig.2012.11.005 PMC3594559

[48] Devaux I, Manissero D, Fernandez de la Hoz K, Kremer K, van Soolingen D, et al (2010) Surveillance of extensively drug-resistant tuberculosis in Europe, 2003–2007. Euro Surveill 15 doi:pii: 19518 20338144

[49] Perdigao J, Macedo R, Malaquias A, Ferreira A, Brum L, et al (2010) Genetic analysis of extensively drug-resistant *Mycobacterium tuberculosis* strains in Lisbon, Portugal. J Antimicrob Chemother 65: 224–227. 20028780 10.1093/jac/dkp452

[50] Niemann S, Diel R, Khechinashvili G, Gegia M, Mdivani N, et al (2010) *Mycobacterium tuberculosis* Beijing lineage favors the spread of multidrug-resistant tuberculosis in the Republic of Georgia. J Clin Microbiol 48: 3544–50–3550. 20702677 10.1128/JCM.00715-10 PMC2953096

[51] Iwamoto T, Yoshida S, Suzuki K, Wada T (2008) Population structure analysis of the *Mycobacterium tuberculosis* Beijing family indicates an association between certain sublineages and multidrug resistance. Antimicrob Agents Chemother 52: 3805–3809. 18694954 10.1128/AAC.00579-08 PMC2565899

